# Outbreak detection and evaluation of a school-based influenza-like-illness syndromic surveillance in Tianjin, China

**DOI:** 10.1371/journal.pone.0184527

**Published:** 2017-09-08

**Authors:** Wenti Xu, Tianmu Chen, Xiaochun Dong, Mei Kong, Xiuzhi Lv, Lin Li

**Affiliations:** 1 Tianjin Centers for Disease Control and Prevention, Tianjin, China; 2 Changsha Center for Disease Control and Prevention, Changsha, China; 3 Hangu Center for Disease Control and Prevention, Binhai New Area, Tianjin, China; University of Calgary, CANADA

## Abstract

School-based influenza-like-illness (ILI) syndromic surveillance can be an important part of influenza community surveillance by providing early warnings for outbreaks and leading to a fast response. From September 2012 to December 2014, syndromic surveillance of ILI was carried out in 4 county-level schools. The cumulative sum methods(CUSUM) was used to detect abnormal signals. A susceptible-exposed-infectious/asymptomatic-recovered (SEIAR) model was fit to the influenza outbreak without control measures and compared with the actual influenza outbreak to evaluate the effectiveness of early control efforts. The ILI incidence rates in 2014 (14.51%) was higher than the incidence in 2013 (5.27%) and 2012 (3.59%). Ten school influenza outbreaks were detected by CUSUM. Each outbreak had high transmissibility with a median R_unc_ of 4.62. The interventions in each outbreak had high effectiveness and all R_con_ were 0. The early intervention had high effectiveness within the school-based ILI syndromic surveillance. Syndromic surveillance within schools can play an important role in controlling influenza outbreaks.

## Background

Syndromic surveillance was developed in the 1990s to detect and respond to bioterrorism events. It now has become an attractive public health tool and has been widely used in influenza control and other fields because of its remarkable ability to adapt to various public health requirements [1]. It is more sensitive and more timely than traditional surveillance methods [2]. Traditional disease surveillance is based on the disease diagnosis, which delays public decisions about outbreaks by one to two weeks [3]. Additionally, syndromic surveillance is well suited to rural communities in developing countries [4].

Syndromic surveillance uses data from clinic visits, hotlines, internet questionnaires, over the counter drug (OTC) purchasing, absenteeism and laboratory testing to provide early alerts about an outbreak of an unspecified disease and provide data for various public health measures. Currently, syndromic surveillance is widely used in the world as a complement to traditional surveillance [5]. Syndromic surveillance can also be used to reduce transmission of most infectious diseases, including emerging infectious diseases. For example, with Ebola, governments monitored villages for signs of disease spread in community, which could detect residual Ebola transmission and future Ebola outbreaks, and could even be used for other infectious diseases [6,7].

Until now, influenza sentinel surveillance has mainly been established in big city hospitals; cases come from different communities or schools, but this type of surveillance has low sensitivity to outbreaks in certain community or schools especially in rural areas. Syndromic surveillance of influenza-like-illness (ILI) in schools can ensure early warning and adequate response capacity, particularly in developing countries or regions, where it is difficult to process laboratory confirmation in a timely manner because of limited local resources [8,9]. Additionally, infectious diseases spread quickly in crowded settings like schools, so school-based ILI syndromic surveillance may be more sensitive to detect outbreaks than other syndromic surveillance methods, such as OTC frequency in drugstores [10]. Currently, school-based syndromic surveillance has been incorporated into community influenza surveillance in China.

We carried out syndromic surveillance in county-level schools by an examination of absenteeism to detect outbreaks within the community. We chose cumulative sum methods (CUSUM) to detect abnormal signals to confirm the ILI outbreak. Specimens from ILI cases were collected to detect the influenza virus. We evaluated the effectiveness of early control efforts directed at these influenza outbreaks on school absenteeism by using a susceptible-exposed-infectious/asymptomatic-recovered (SEIAR) model. The study reports our experience in carrying out this syndromic surveillance in schools.

## Methods

### Ethics statement

ILI data collection from students was considered public health surveillance by the National Health and Family Planning Commission of the People’s Republic of China. Verbal informed consent was obtained from all individuals enrolled in this study. Swabs from minors were only obtained after written, informed consent was obtained from their parents or legal guardians. Surveillance and sampling protocols were approved by the biomedical ethical review committee of the Tianjin Centre for Disease Control and Prevention and complied with the Helsinki Declaration of 1975, as revised in 2008.

### Study area

We conducted the study at 4 schools in Hangu county, Binhai New Area, Tianjin, China, from September 1, 2012, to December 31, 2014. The ILI incidence rate (ILI%) in local sentinel surveillance hospitals from September to December 2012 was 1.99%, and ILI% increased in 2013 (2.14%) and in 2014 (2.75%). We selected 3 primary schools from a total of 6 schools and 1 high school from a total of 2 high schools in this area. The number of students per school ranged from 1247 to 1798, with a total of about 6000 students in all 4 schools.

### Data source and collection

Continuous monitoring of school absentees was carried out in the 4 schools. The administered questionnaire was designed to record detailed information on students’ absences due to sickness, including date of absence, the basic information on the students, the cause of the absence, the disease or syndrome (fever including body temperature, cough, and/or sore throat), and onset date. The questionnaire was administered by teachers, who checked if the students were absent, and called their parents to ask the reason for the absence. The teachers reported the questionnaire to the school doctor in the morning. The school doctor immediately input that information into computer and emailed the Tianjin Centre for Disease Control and Prevention (CDC). ILI was defined as fever (≥ 100.4°F or 38°C) and cough or sore throat. The ILI incidence rate (ILI%) was defined as the total ILI cases divided by the total number of students.

To verify validity of the data, local CDC workers checked the data every day, and selected some sick students and called their parents, and compared the information with that on the reported questionnaires.

### Early alerts of influenza-like illness outbreak based on cumulative sum

CDC workers analyzed the data daily and judged whether an outbreak of ILI occurred using CUSUM [7]. We used CUSUM control charts for ILI% to detect deviations from expected values. CUSUM can use C1, C2 and C3 algorithms, which have different sensitivities to detect aberrations: C1 is the least sensitive and C3 is the most sensitive. The C1 algorithm is defined as the current ILI case count over the past 7 days being ≥3 standard deviations (SD) greater than the mean. The C2 algorithm is the same as C1, except the time period is shifted by 2 days between the baseline and current day being evaluated. The C3 algorithm calculates a partial sum for the last three days which have a positive value over the mean; like C2, the baseline mean and SD are based on the past 7 days ILI shifted by 2 days. The aberration detection charts display a “C3” signal when CUSUM values exceed 2 [8]. The equation of CUSUM calculation can be written as follows [9]:

*S*_*t*_ = max(0; *S*_*t*-1_ + ((*χ*_*t*_ − (*μ*_0_ + *kσ*_*χt*_))/*σ*_*χt*_)) with a decision value of *S*_t_>2, where *χ*_*t*_ is the ILI, *μ*_0_ is the expected value, *σ*_*xt*_ is the standard deviation, *k* is the detectable shift from the mean, *S*_t_ is the current CUSUM calculation, and *S*_t − 1_ is the previous CUSUM calculation[11]. The outbreaks of ILI were confirmed if CUSUM gave two continuous C3 alarms in the school-based syndromic surveillance system within one week.

### Pathogen test of influenza

Throat swab specimens of ILI patients were collected by the local CDC workers after confirmation of the ILI outbreak confirmation. Specimens were stored at 4°C and transported to the influenza network laboratory within 48 h by local CDC staff. RT-PCR was used to subtype respective influenza strains.

### Evaluate early control measure effectiveness

#### Model with no intervention

A susceptible-exposed-infectious/asymptomatic-recovered (SEIAR) model is suitable for simulating influenza transmission [12,13]. The model is expressed by differential equations as follows:

$$\left\{ \begin{array}{l} dS/dt=-\beta S(I+\kappa A)\\ dE/dt=\beta S(I+\kappa A)-p\omega^{\prime }E-(1-p)\omega E\\ dI/dt=(1-p)\omega E-\gamma I\\ dA/dt=p\omega^{\prime }E-\gamma^{\prime }A\\ dR/dt=\gamma I+\gamma^{\prime }A \end{array}\right.$$ (1)

In these equations, *S*, *E*, *I*, *A*, and *R* refer to susceptible, exposed, symptomatic, asymptomatic, and removed individuals, respectively. *dS/dt*, *dE/dt*, *dI/dt*, *dA/dt*, and *dR/dt* refer to time *t* and the changing rates of the *S*, *E*, *I*, *A*, and *R* populations, respectively. *β*, *ω*, *ω′*, *γ*, *γ′*, *κ*, and *p* refer to transmission relative rate, incubation period relative rate, latent period relative rate, removal rate parameter of symptomatic individuals, removal rate parameter of asymptomatic individuals, parameter for the transmission rate of asymptomatic compared with symptomatic individuals, and proportion of asymptomatic infections, respectively (Table 1).

**Table 1:** Parameter definitions and values within the SEIAR model.

Parameter	Description	Unit	Value	Range	Method
*β*	Person—to-person contact rate	1	See text	0–1	Curve fitting
*k*	Relative transmissibility rate of asymptomatic to symptomatic individuals	1	0.5	0–1	References[12,13,19]
*ω*	Incubation relative rate	day^-1^	0.5263	0.1429–1	References[1 2,13,19]
*ω′*	Latent relative rate	day^-1^	0.8333	0.1429–1	References[12,13,19]
*p*	Proportion of the asymptomatic	1	0.14	0–1	References[12]
*γ*	Recovery rate of the infectious	day^-1^	0.2342	0.0833–1	References[12]
*γ′*	Recovery rate of the asymptomatic	day^-1^	0.2439	0.0714–1	References[12,13,19]

#### Reproduction number

Reproduction numbers with and without control measures, defined as *R*_unc_ and *R*_con_, were employed to know the effectiveness of the interventions in each outbreak. The reproduction number (*R*) is defined as the expected number of secondary infections that result from introducing a single infected individual into an otherwise susceptible population [12–17]. If *R*<1, the number of infected individuals would decrease toward zero, and the disease would therefore be gradually eliminated. In contrast, if *R*>1, the disease would become more prevalent. According to the definition of *R* and the methods reported by Chen et al. [12]. and Arino et al. [18], the *R* expression in “model (1)” is as follows:

$$R=\beta S\left(\frac{1-p}{\gamma }+\frac{kp}{\gamma^{\prime }}\right)$$

#### Estimation of parameters

**Fig 1:**
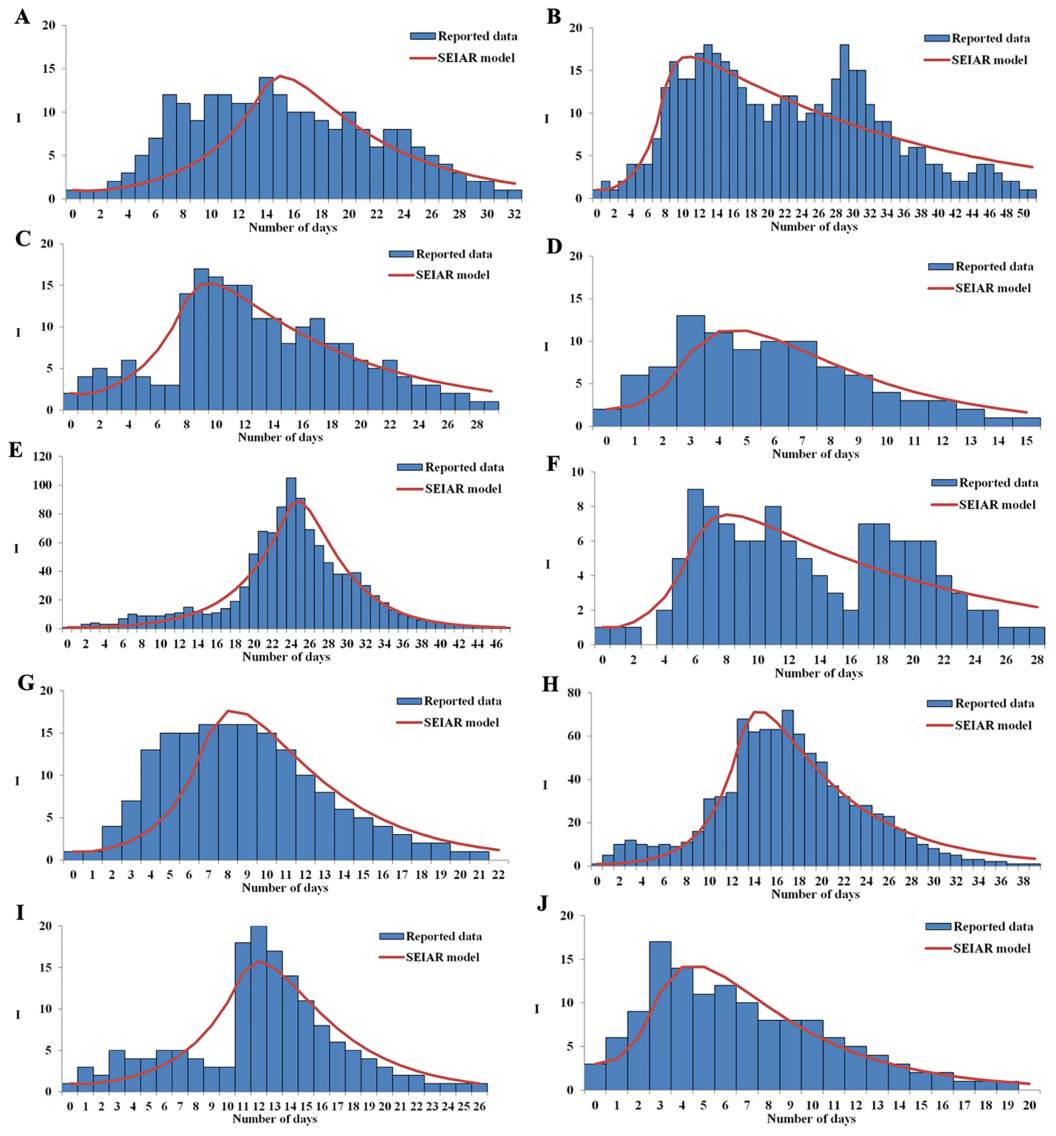
Effectiveness of early interventions during ten influenza outbreaks according to an SEIAR model.

Table 1 shows the parameter definitions and values within the SEIAR model. The results of previously published studies showed that the mean incubation period of influenza was 1.9 days (range 1–7 days), the mean latent period 1.2 days, the mean infectious period 4.1 days, and asymptomatic people were half as infectious as those with influenza symptoms [12,13,19]. The average proportion of asymptomatic influenza in small-scale outbreaks was 0.14 [12]. Thus *ω* = 0.5263, *ω′* = 0.8333, *γ′* = 0.2439, *k* = 0.5 and *p* = 0.14. The removal rate of symptomatic individuals is *γ*, which is the reciprocal of the duration of illness from onset to recovery, which was obtained from a previously published study [12], where *γ* = 0.2342. The parameter *β* was solved by fitting a curve on a typical outbreak as shown in Fig 1.

### Simulation methods

Berkeley Madonna 8.3.18 (University of California at Berkeley, Berkeley, USA) and Microsoft Office Excel 2010 (Microsoft, Redmond, USA) software were employed for model simulation and figure development, respectively. The details of model fitting methods run in Berkeley Madonna, such as Runge-Kutta method of order 4 and root-mean-square deviation, were the same as the ones in previous literature [12–17,20].

## Results

### Detected influenza outbreaks based on CUSUM

The surveillance in the four schools was carried out from September 2012 to December 2014(S1 File). The actual surveillance was 633 days; 219 days of summer and winter vacation were not included in the dataset. We counted the ILI case according to the time of onset, and not the date of absence because the latter method will be zero during weekends and holidays. The daily average ILI case was 1 during the monitoring periods (Table 2).

**Table 2:** Median days of ILI sickness and ILI incidence rate in four schools.

Surveillance school	Type of school	Total number of students			Median daily number with ILI (range)			ILI incidence rate (%)		
		2012	2013	2014	2012.9–12	2013	2014	2012.9–12*	2013	2014
A	Primary	1438	1516	1534	2(0,10)	1(1,5)	2(0,12)^a^	8.97	6.93	12.13
B	Primary	1713	1683	1798	1(0,7)	1(1,5)	2(0,40)^a^	1.81	6.24	16.35
D	Primary	1247	1397	1356	1(0,5)	0(0,8)	3(1,42)^a^	2.57	3.22	20.28
C	Middle	1454	1644	1485	0(0,5)	1(0,5)	1(0,16)^a^	1.24	4.56	9.16
Total		5852	6258	6139				3.59^§^	5.27^§^	14.51^§^

ILI: Influenza-like illness

*Quarterly incidence rate

^§^ILI Incidence rate in 2014 was higher than that in 2013 and September to December 2012. (*χ*^2^ = 298.02, p = 0.00001; *χ*^2^ =, p = 0.00001)

^a^ The high absence occurred during an influenza outbreak.

The ILI incidence rates (Table 2) in 2014 (14.51%) were higher than that in 2013 (5.27%) and September to December 2012 (3.59%).

**Fig 2:**
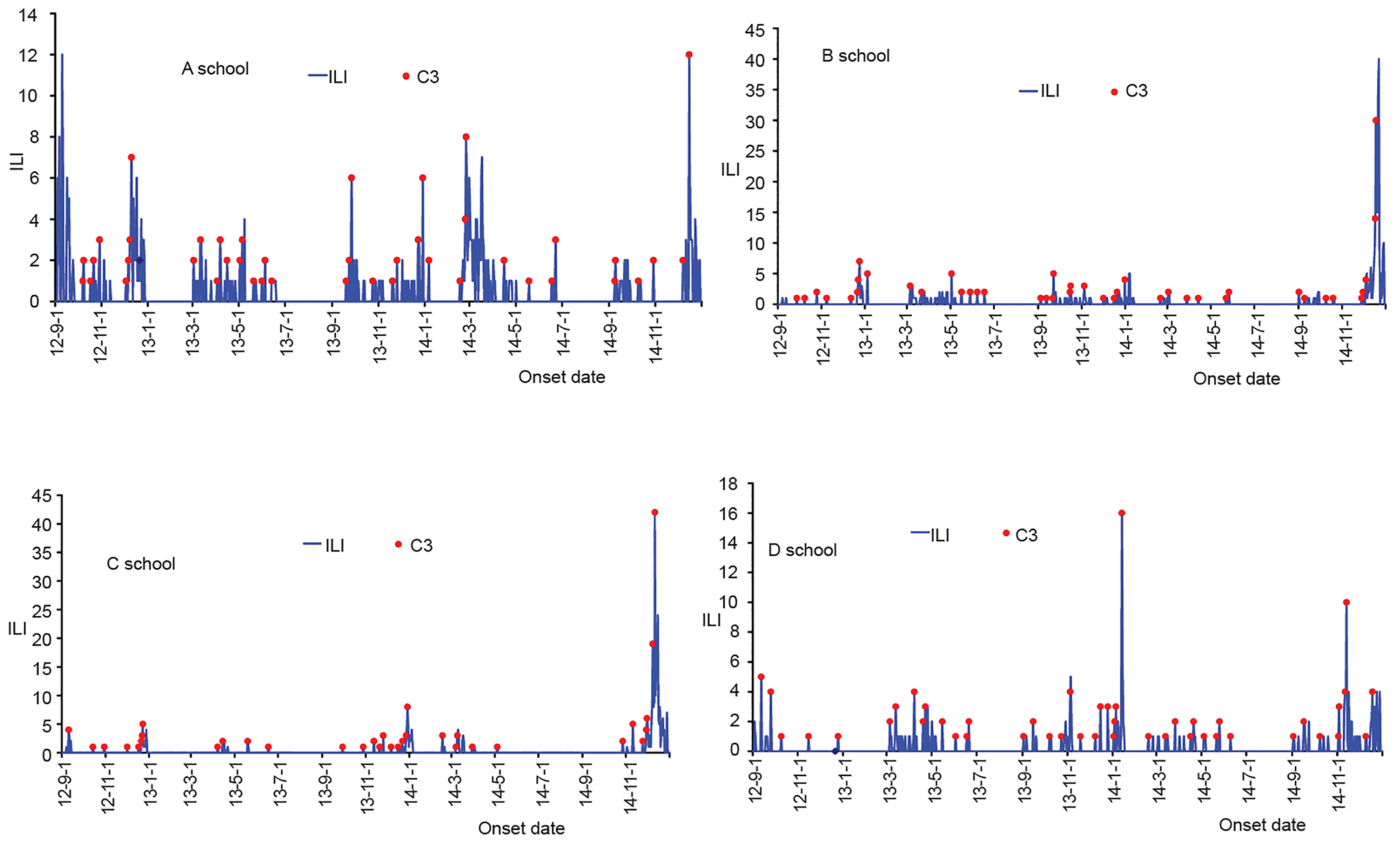
Ten influenza outbreaks within four schools detected by CUSUM.

Ten influenza outbreaks of the four schools were detected by CUSUM (Fig 2). Among of them, three outbreaks were reported in 2012, and seven in 2014. Seven outbreaks were caused by A (H3N2), and other three were 2009 A (H1N1) (Table 3). We took control measures according to the outbreak given by CUSUM, such as isolating the cases for three days, disinfecting the environment, and opening the window, et al.

**Table 3:** Information and reproduction numbers of each ILI outbreak.

Outbreak ID	Reported time	Influenza type	Total cases	Affected students	Attack rate %	*R*_*unc*_	*R*_*con*_
1	2012/12/17	A(H3N2)	38	1438	2.64	2.67	0.00
2	2014/2/25	2009 A(H1N1)	36	1534	2.35	4.78	0.00
3	2014/12/15	A(H3N2)	43	1534	2.8	4.02	0.00
4	2012/12/25	A(H3N2)	50	1713	2.92	9.45	0.00
5	2014/12/5	A(H3N2)	60	1798	3.34	2.77	0.00
6	2012/12/25	A(H3N2)	27	1247	2.17	4.85	0.00
7	2014/1/2	2009 A(H1N1)	118	1356	8.7	5.83	0.00
8	2014/12/12	A(H3N2)	45	1397	3.22	4.46	0.00
9	2014/1/14	2009 A(H1N1)	27	1644	1.64	3.60	0.00
10	2014/11/15	A(H3N2)	30	1485	2.02	8.55	0.00

### Reproduction number and the effectiveness of interventions

Fitting a curve to the SEIAR model and the 10 outbreaks were shown to have no statistical significance by a Chi square test (*χ*^2^< 0.15, P> 0.05) (Fig 1). The results of the curve fitting read that each outbreak had high transmissibility with a median R_unc_ of 4.62 (ranging from 2.67 to 9.45). The interventions we employed in each outbreak were highly effective, with a R_con_ of 0 (Table 3).

## Discussion

This study detected abnormal signals by CUSUM in the school-based ILI syndromic surveillance, and a SEIAR model was fit to estimate a counterfactual influenza outbreak without control measures, which was then compared with the actual influenza outbreak to evaluate the effectiveness of early control efforts. Ten school influenza outbreaks were detected by CUSUM from September 2012 to December 2014. Each outbreak had high transmissibility with a median R_unc_ of 4.62. Our study shows that the early intervention had high effectiveness and all R_con_ were 0. Syndromic surveillance within schools can play an important role in controlling influenza outbreak.

Our study found that the ILI incidence rates in 2014 (14.51%) were higher than that in 2013 (5.27%) and September to December 2012 (3.59%). Another study has found that A(H3N2) activity in China has increased since 2012, when group 3C virus was predominant; group 3C divided into group 3C.3a in beginning of 2014 and group 3C.2a during the end of 2014 and the beginning of 2015 [21]. People were easily infected by group C3C.2a, and 5 influenza outbreaks at the end of 2014 were caused by A(H3N2). The big outbreaks in 2014 could have increased the sensitivity of ILI reports and the rapid response to outbreaks in schools.

The big outbreaks at the end of 2014 mainly (72%) happened at the primary schools, among children 7–13 years of age. Vaccination coverage in this age group was only 3.67%. Moreover, vaccine effectiveness was low during the 2014/2015 influenza season because of vaccine mismatch [21].

For outbreak ID#4, the attack rate of influenza was 2.92% but R_unc_ was 9.45. This discrepancy could have resulted because teachers reported substantial absenteeism on Friday and symptomatic students were separated on the first day of outbreaks. Subsequently, the schools closed for the weekend. All these control measures effectively limited the spread of the outbreak.

ILI syndromic surveillance in schools is one possible mechanism to surveillance for community influenza. Other community influenza surveillance mechanisms include ILIs in clinic visits and pharmacy based methods, like frequency of purchasing OTC drugs. School syndromic data can detect increased influenza transmission in a timely manner [22], and showed better performance of outbreak detection compared with clinic methods and OTC drug purchases [10]. Influenza outbreaks within schools imply transmission of influenza throughout the community because students are connected to the community and schools. The detected outbreaks showed real-time abnormalities in influenza incidence by time and place. Several other studies have evaluated the effectiveness of interventions using simulated data, This study evaluated the performance of early intervention using real outbreak data from school syndromic surveillance, which provides a higher degree of real-world information than simulated data [23,24].

CUSUM can be used to specify thresholds based on short-term data. The daily surveillance data in schools were used to give a prospective alarm, which is quicker to generate an alarm and enables immediate control measures. In this study, we found that two successive C3 signals can be a signal for a real ILI outbreak, and but only one C3 signal may be a false positive and does not detect an outbreak with high validity [25,26]. Early intervention measures will not only decrease the number of ILI case and the absenteeism rate for students by mitigating the spread between students, but also reduce the cost of control measures [27].

A SEIAR model was used to evaluate the effectiveness of the early interventions that used the school-based syndromic surveillance. We compared the reproduction numbers of the influenza outbreak with and without control measures after the CUSUM-issued warning signals. The median R_unc_ in 10 influenza outbreaks was 4.62, and the highest R_unc_ was 9.45 caused by the H3N2 influenza subtype. The R_con_ was near zero after an early intervention based on CUSUM in this study. This number is lower than in a similar study which took control measures after an outbreak defined as 30 ILIs [17]. In this study, we took daily data based on onset date, which can decrease the effect of seasonal influenza periods and weekend effect. But the low daily case numbers of ILI cases in the syndromic surveillance system can affect detection of aberrations, especially in the influenza off-season. Some studies have used weekly ILI data to detect the influenza outbreaks, which does not allow for a timely response, and which delays the best control time, and which led to further spread of influenza in the school. In the future, we can take daily absenteeism data with more numbers to give a first alarm, and then identify the ILI syndrome to detect the outbreak based on CUSUM. Additionally, we also could establish electronic syndromic surveillance systems in schools to detect influenza outbreaks in real time.

## Supporting information

S1 File Raw data. Data file for one outbreak in a school.
(XLSX)
